# Effect of quorum quenchers on virulence factors production and quorum sensing signalling pathway of non-mucoid, mucoid, and heavily mucoid *Pseudomonas aeruginosa*

**DOI:** 10.1007/s11274-022-03339-9

**Published:** 2022-07-15

**Authors:** Rachith Kalgudi, Roya Tamimi, Godfrey Kyazze, Tajalli Keshavarz

**Affiliations:** School of Life Sciences, University of Westminster, 115 New Cavendish Street, W1W 6UW London, UK

**Keywords:** Microbial biofilm, Pathogenicity, Bacterial communication.

## Abstract

Quorum quenching (QQ), a mechanism which inhibits, interferes or inactivates quorum sensing, has been investigated for control of biofilms instigated by quorum sensing process. Application of quorum quenchers (QQs) provides the possibility to investigate how different phenotypes of *Pseudomonas aeruginosa* (non-mucoid, mucoid, and heavily mucoid strains) modulate their gene expression to form biofilms, their quorum sensing (QS) mediated biofilm to be formed, and their virulence expressed. The mRNA expression of the AHL-mediated QS circuit and AHL-mediated virulence factors in *P. aeruginosa* was investigated in presence of QQs. qPCR analysis showed that farnesol and tyrosol actively reduce the expression of the synthase protein, LasI and RhlI, and prevent production of 3OC12-HSL and C4-HSL, respectively. Also, the use of farnesol and tyrosol significantly moderated gene expression for exo-proteins *toxA, aprA, LasB*, as well as *rhlAB*, which are responsible for rhamnolipid production. Our findings were promising, identifying several suppressive regulatory effects of furanone and *Candida albicans* QS signal molecules, tyrosol, and farnesol on the AHL-mediated *P. aeruginosa* QS network and related virulence factors.

**Supplementary Information:**

The online version contains supplementary material available at 10.1007/s11274-022-03339-9.

## Introduction

In humans, *Pseudomonas aeruginosa* strains can cause infections and diseases such as wound infections, periodontitis, keratitis and chronic pneumonia during cystic fibrosis (CF). These usually occur in individuals with compromised immune systems (Gellatly and Hancock 2013). The diversity of genetic variation amongst *P. aeruginosa* strains is highlighted by the presence of non-mucoid, mucoid and heavily mucoid phenotypes (Workentine et al. 2013).

An important factor contributing toward the pathogenesis of *P. aeruginosa* is its remarkable ability to switch between planktonic and sessile modes of growth in the onset of chronic infections. The sessile cells, compared to planktonic cells, do not show an enhanced resistance towards antibiotics. However, they show an increased threshold in their minimum inhibitory concentration (MIC) against the antibiotic of interest when compared as they are enveloped within an extracellular matrix (Lebeaux et al., 2014). The basic assumption regarding the protective role played by the biofilm is that of reduced diffusion of antibiotics into the inner layers of the biofilms and their effective access to the sessile bacteria (Mah et al. 2003; Singh et al. 2021).

*P. aeruginosa* produces numerous virulence factors that aid in its colonisation during an infection. Virulence factors such as mucoid exopolysaccharides, rhamnolipids, haemolysins, proteases, lipopolysaccharides, pili, and lipases are common tools used by *P. aeruginosa* to invade, adhere and colonise the host. The arsenal of virulence factors makes *P. aeruginosa* a potent pathogen (Schaber et al. 2004; Schroeder et al. 2017).

Many processes involving virulence are modulated by the hierarchical quorum sensing (QS) circuits documented in *P. aeruginosa*. These traits are involved in the pathogenicity of the bacteria (Lee and Zhang 2014). It has been proposed that targeting the QS system of *P. aeruginosa* by interrupting bacterial communication instead of killing the bacteria by antibiotics would have an anti-pathogenic effect and may aid in the fight against biofilm forming pathogens and antibiotic resistance (Barlow and Nathwani 2005; Rémy et al. 2020).

Conventional *P. aeruginosa* antibiotics such as ceftazidime, ciprofloxacin, and azithromycin display QQ activity (Skindersoe et al. 2008; Swatton et al. 2016). However, even though the mechanism is not fully understood, it has been speculated that their QQ activity involves inhibiting bacterial protein synthesis which prevents the expression of the inducer protein from synthesising the QS signal molecules (Reuter et al., 2016). Azithromycin is also known to inhibit alginate production in mucoid strains of *P. aeruginosa* (Imperi, Leoni and Visca, 2014). In a recent study, the newly synthesised *P. aeruginosa* quorum-sensing autoinducer analogues (AIA-1, -2) were demonstrated to boost the bactericidal efficacy of azithromycin by altering the cell surface hydrophobicity of *P. aeruginosa*, reducing antibiotic tolerance (Abe et al. 2021). The use of the said antibiotics has been known to induce resistance amongst bacteria, hence they cannot be used as potential QQs.

As Acyl-Homoserine Lactones (AHLs) represent the primary QS signal molecules in *P. aeruginosa*, utilising small molecules that mimic AHLs to inhibit QS is a promising strategy. Furanones were first identified as QQs that inhibit by mimicking AHL molecules by attaching to the LasR receptor of *P. aeruginosa*. This interfered with the binding of the AHL molecule, thereby preventing QS mediated gene regulation in *P. aeruginosa* (Manner and Fallarero., 2018). Since the discovery of the role of furanone in inhibition of the QS system, numerous synthetically produced, structurally diverse furanone derivatives have been synthesised (Irie et al. 2017). Similarly, an AHL analogue, meta-bromo-thiolactone, competitively inhibits QS in *P. aeruginosa* and prevents biofilm formation and pyocyanin production and protects lung cells against the antagonistic activity of *P. aeruginosa* (O’Loughlin et al., 2013).

While there are numerous environmental and physiological cues for dispersal of biofilm, chemicals produced by microorganism themselves can be utilised in a strategy to induce biofilm dispersal and inhibit QS in *P. aeruginosa*. *Candida albicans* produces two QS molecules, namely farnesol and tyrosol (Kaplan 2010). Farnesol was shown to inhibit the morphological shift from yeast to hyphae at high cell densities while tyrosol was shown to accelerate the transition from yeast to hyphae in *C. albicans* (Decanis et al. 2011). As *P. aeruginosa* and *C. albicans* are known to coexist in numerous nosocomial opportunistic infections (Méar et al. 2013; Doing et al. 2020), the influence of farnesol and tyrosol as potential QQs may be investigated against *P. aeruginosa* biofilm formation and subsequent virulence production between the non-mucoid and mucoid phenotypes along with other QQs and biofilm dispersal agents.

This study investigated the effect of quorum quenching (QQ) on biofilm formation and virulence factor secretion of three strains of *P. aeruginosa*: *P. aeruginosa* NCTC 10,662 (non-mucoid), *P. aeruginosa* PAO1 (mucoid) and *P. aeruginosa* RBHi (a heavily mucoid CF isolate).

## Materials and methods

### Preparation of bacterial inoculum, biofilm formation and dispersal

*P. aeruginosa* NCTC 10,662 (non-mucoid), *P. aeruginosa* PAO1 (semi mucoid) and *P. aeruginosa* RBHi (heavily mucoid) were selected as model phenotypes for this study.

*P. aeruginosa* PAO1 and *P. aeruginosa* NCTC 10,662 were obtained from the University of Westminster, London culture collection. *P. aeruginosa* CF isolate was kindly donated by the culture collection facility at Royal Brompton Hospital, London, UK (referred to as RBHi in this study).

*P. aeruginosa* strains were subcultured into Lysogeny broth (LB broth) from the slants and incubated aerobically overnight (16–18 h) at 37^o^C at 180 rpm. The absorbance (OD_600_) of the overnight growth of *P. aeruginosa* sp. was readjusted in sterile LB broth to obtain an equivalence absorption according to 0.5 McFarland standards (~ 1.5 × 10^8^ cells) and used for further biofilm growth. A flow-cell (Transmission Flow-cell, FC 281, BioSurface Technologies Corporation (BST), Montana, USA) provided a closed system where bacterial attachment occurred and biofilm structure and hence extracellular polymeric substances (EPS) were produced.

Quorum quenchers used in this study included (Z-)-4-Bromo-5-bromomethylene)-2(5 H)-furanone; E, E – farnesol; and 2,4- hydroxyphenyl)-ethanol (Tyrosol). Equation 1 was used to calculate the MIC50s, concentrations that inhibited 50% of the isolates, for farnesol, tyrosol, and furanone (Quave et al. 2008). A mean of 3 absorbance readings were taken at OD_595_ nm for each value (n = 3).

Eq. 1 $$\scriptsize (1-\left(ODs24-ODs0)\diagup(ODc24-ODc0\right))\times 100=MIC50\left(\%\right)$$

ODs24 = Optical density of culture at 595 nm of experimental sample, 24 h post-inoculation.

ODs0 = Optical Density of culture at 595 nm of experimental sample at time point 0.

ODc24 = Optical density of culture at 595 nm of control sample, 24 h post inoculation.

ODc0 = Optical density of culture at 595 nm of control sample at time point 0.

MIC_50_ were 125 µg/mL for farnesol and tyrosol, and 5 µg/mL for furanone.

1. Farnesol 125 µg/mL (MIC50).

(1-(1.01–0.51/ 1.59 − 0.56)) x 100 = 51.5%

2) Tyrosol 125 µg/mL (MIC50).

(1-(0.94 − 0.48/ 1.59 − 0.56)) x 100 = 55.4%

3) Furanone 5 µg/mL (MIC50).

(1-(0.89 − 0.45/ 1.59 − 0.56)) x 100 = 57.3%

**qPCR analysis of QS network and virulence factors in*P. aeruginosa***.

RNA extraction from bacterial cells was performed using the Trizol RNA isolation protocol described by Chomczynski and Sacchi in 1987. cDNA preparation was then done by using the QuantiTect Reverse Transcription kit (Qiagen ltd, Manchester, UK).

Samples were prepared using diluted forward and reverse primer (Tables 1 and 2) mix (1/10 dilution). They were then run in duplicate at 95 °C initial denaturation for 2 min. Subsequently, the amplification program involved 40 cycles of denaturation at 95 °C for 15 s, primer annealing at 55 °C for 15 s and extension at 72 °C for 30 s. A final extension was performed at 72 °C for 2 min followed by cooling at 4 °C. A dissociation step at 55 °C was used to generate a melting curve with a 1 °C increase every 5 s till 95 °C to obtain verification of amplified product. Reference genes were as reported in Table 3. SYBR Green qPCR was performed using Rotor Gene Q (Qiagen ltd, Manchester, UK).

**Table 1:** Primers used in this study for QS genes in *P. aeruginosa*

Gene	Primer	Nucleotide sequence	Reference
*lasI*	forward	5’ CGTGCTCAAGTGTTCAAGG 3’	Jack et al. 2018; Zhu et al. 2004
	reverse	5’ TACAGTCGGAAAAGCCCAG 3’	
*lasR*	forward	5’ AAGTGGAAAATTGGAGTGGAG 3’	
	reverse	5’ GTAGTTGCCGACGACGATGAAG 3’	
*rhlI*	forward	5’ TTCATCCTCCTTTAGTCTTCCC 3’	
	reverse	5’ TTCCAGCGATTCAGAGAGC 3’	
*rhlR*	forward	5’ TGCATTTTATCGATCAGGGC 3’	
	reverse	5’ CACTTCCTTTTCCAGGACG 3’	

**Table 2:** Primers used in this study for virulence factors of *P. aeruginosa*

Gene	Primer	Nucleotide sequence	Reference
*toxA*	forward	5’ GGAGCGCAACTATCCCACT 3’	Sabharwal et al. 2014; Aghamollaei et al. 2015
	reverse	5’ TGGTAGCCGACGAACACATA 3’	
*aprA*	forward	5’ GTCGACCAGGCGGCGGAGCAGATA 3’	
	reverse	5’ GCCGAGGCCGCCGTAGAGGATGTC 3’	
*rhlAB*	forward	5’ TCATGGAATTGTCACAACCGC 3’	
	reverse	5’ ATACGGCAAAATCATGGCAAC 3’	
*lasB*	forward	5’ TTCTACCCGAAGGACTGATAC 3’	
	reverse	5’ AACACCCATGATCGCAAC 3’	

**Table 3:** Reference genes and primers for qPCR

Gene	Primer	Nucleotide sequence	Reference
*rpsL*	forward	5’ CCTCGTACATCGGTGGTGAAG 3’	Pourakbari et al., 2015; Jack et al. 2018
	reverse	5’ CCCTGCTTACGGTCTTTGACAC 3’	
*AmpC*	forward	5’GGTGCAGAAGGACCAGGCACAGAT 3’	
	reverse	5’CGATGCTCGGGTTGGAATAGAGGC 3’	

### Statistical analysis

All experiments were performed in triplicates. All data for assays performed in this study were statistically analysed using GraphPad Prism to determine *p* values and establish correlation between data sets. All graphs were plotted using GraphPad Prism. p < 0.05 was considered significant.

## Results

**Quantitative PCR and expression levels of QS genes and QS mediated virulence factors of*P. aeruginosa***.

**Fig. 1:**
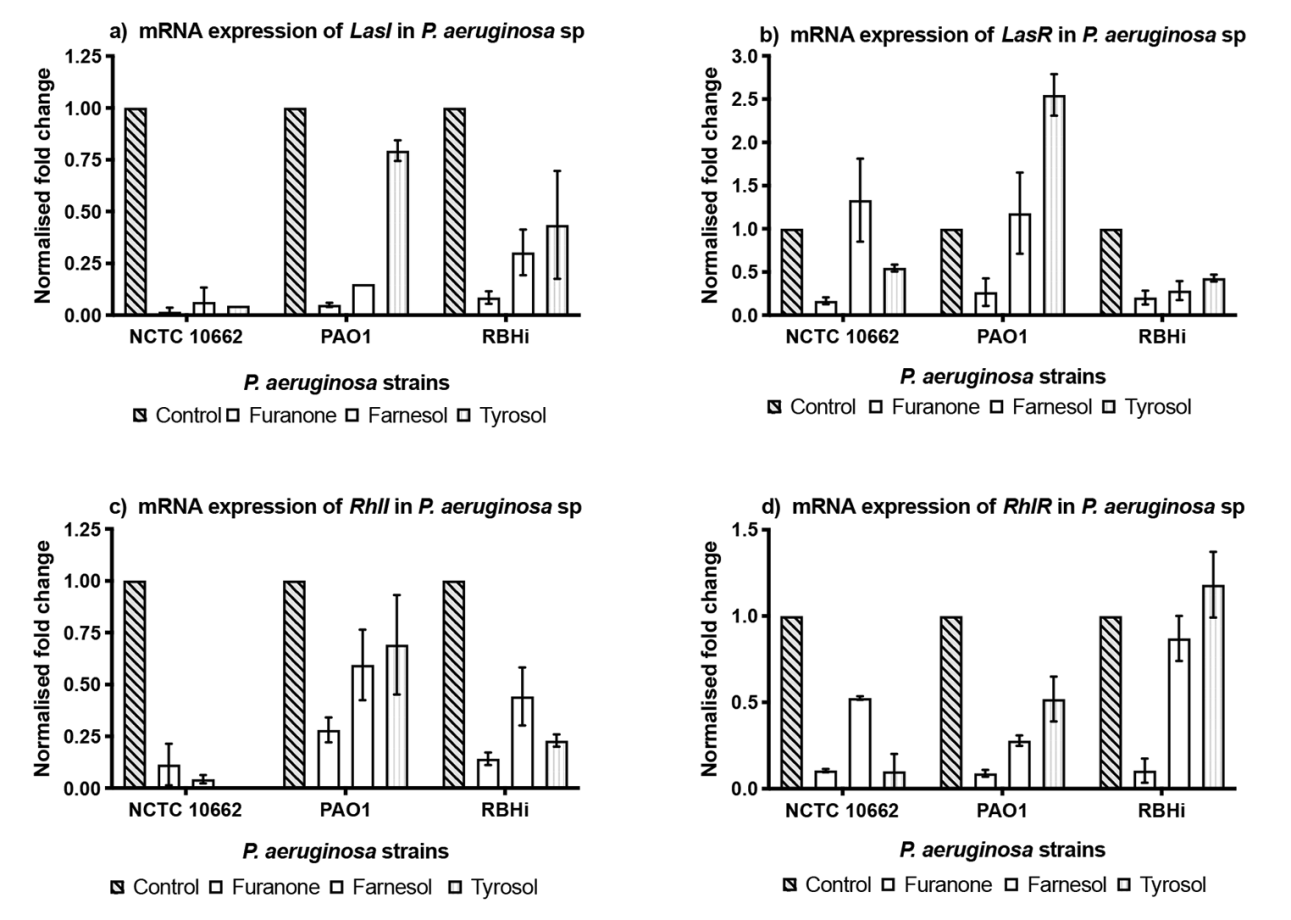
mRNA expression of AHL mediated QS circuit in *P. aeruginosa* sessile cells grown with QQ. The fold change of mRNA for LasI (a), LasR (b), RhlI (c) and RhlR (d) was determined for *P. aeruginosa* sessile cells extracted from biofilm treated with furanone, farnesol and tyrosol after ~ 16 h growth. Results are expressed as the mean fold change (control standardised to 1.0) with error bars representing SEM (n = 9)

Following the treatment with MIC_50_ furanone, farnesol and tyrosol, there was a significant reduction (*p* = 0.0001) in the relative abundance of RNA for all the *LasI* protein (Fig. 1a) from the three strains of *P. aeruginosa*. Comparison between farnesol and tyrosol showed that farnesol had a greater effect on *LasI* compared to tyrosol. The effect of farnesol and tyrosol on *LasI* was more pronounced in NCTC 10,662 compared to PAO1 and RBHi. This was not the case with *LasR*. With the exception of RBHi, treatment with farnesol up-regulated the expression of *LasR* in NCTC 10,662 and PAO1. *LasR* was also up-regulated with tyrosol in PAO1 (Fig. 1b).

Reduction in mRNA expression for *RhlI* protein was seen with all the strains when treated with furanone, farnesol, and tyrosol (Fig. 1c). Similar to *LasI*, NCTC 10,662 showed the highest reduction in RNA content for *RhlI*, while all three strains showed a down-regulation of *RhlI* when treated with farnesol and tyrosol (Fig. 1c). In the case of RBHi, tyrosol up-regulated the expression of *RhlR* (Fig. 1d), however, down-regulation of *RhlR* was marginal when treated with farnesol.

Figure 1.

**Fig. 2:**
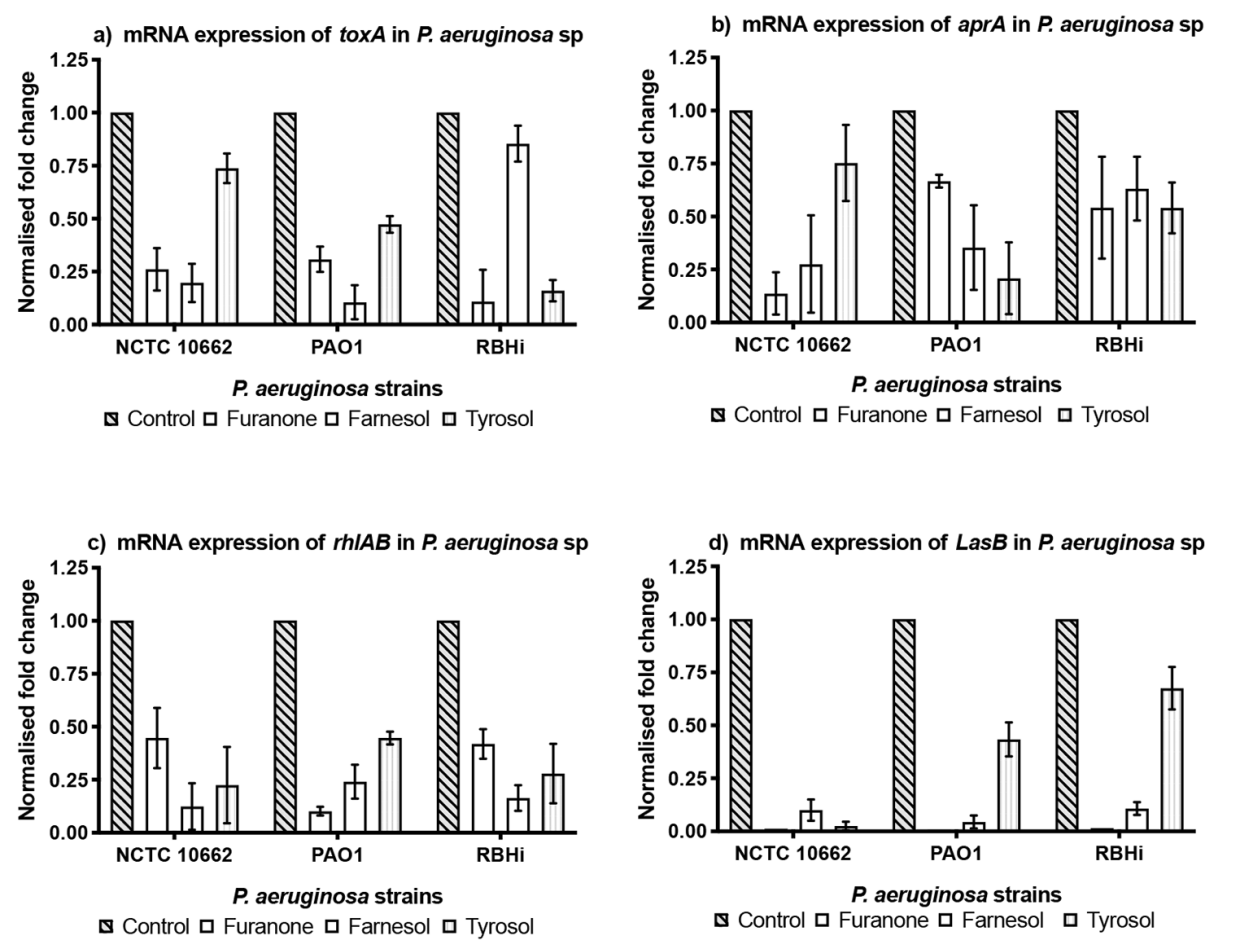
mRNA expression of AHL mediated virulence factors of *P. aeruginosa* sessile cells grown with QQs. The fold change of mRNA for toxA (a), aprA (b), rhlAB (c) and LasB (d) was determined for *P. aeruginosa* sessile cells extracted from biofilm treated with furanone, farnesol and tyrosol after ~ 16 h growth. Results are expressed as the mean fold change (control standardised to 1.0) with error bars representing SEM (n = 9)

qPCR analysis of expression of genes related to virulence factors of *P. aeruginosa* showed that treatment with furanone, farnesol, and tyrosol down-regulated the expression of *toxA, aprA, rhlAB* and *LasB* when compared to the control as shown in Fig. 2. Farnesol seemed highly effective in down-regulating *rhIAB* when compared to tyrosol for all the strains of *P. aeruginosa* (Fig. 2c). *LasB* showed the highest down-regulation amongst all the genes analysed with furanone treatment (Fig. 2d).

Data pertaining to cell counts from the control and experimental cultures that underwent treatment with furanone, farnesol and tyrosol is provided as supplementary information to corroborate the regulation of gene expression in *P aeruginosa* sp.

## Discussion

In recent years, QS has been the primary focus of research involving treatment of biofilm mediated chronic infections. It has been well established that QS plays a vital role in development and formation of biofilms which is a recalcitrant mode of growth and aids in the onset of bacterial resistance towards conventional antibiotics (Pletzer et al. 2018). This has led to an inexorable rise in formation of superbugs-related infections that are extremely hard to treat due to the dearth of effective therapies (Fernández et al. 2011). Therefore, recent research has been focused on elucidating novel therapeutic strategies in order to combat biofilm-related infections by attenuating the ability of cell-to-cell communication by targeting the QS signalling system present in bacteria (Römling and Balsalobre., 2012 and Bi et al. 2021). This in turn would aid in arresting biofilm formation and biofilm related chronic infections. Novel therapies that target the QS system in pathogenic bacteria could provide the foundation for the development of next generation anti-virulence therapies.

Three primary strategies can be adopted to combat biofilm formation by inhibiting QS. The most frequently studied strategies are degradation and modification of QS signals to prevent bacterial communication along with competitive inhibition of the receptor protein of the QS circuit as well as impeding the function of synthase protein responsible for producing the QS molecules (LaSarre and Federle., 2013, Bhatt et al. 2021). In the natural environment, blocking communication of ecological niche adversary is essential for survival. This is a promising avenue to explore in order to get a better understanding of the process of social interaction of individual bacterial species or of groups aiming to dictate the niche (Bhagirath et al. 2016). As vital QS is for bacterial coordination and survival, it is also essential for bacteria to interfere with QS of other microbes in order to gain an advantage for survival (Li and Tian., 2012). This naturally existing process of communication interference to gain an advantage over competitors can be exploited to develop novel therapies targeting the QS system of pathogenic bacteria.

Gene expression studies, involving the individual effect of farnesol and tyrosol, were conducted along with furanone. All the three strains showed a significant down-regulation in both the AHL mediated synthases (*LasI* and *RhlR*) when treated with furanone (Fig. 1). However, *LasR* receptor was up-regulated in NCTC 10,662 using farnesol and in PAO1 using farnesol and tyrosol. Similar up-regulation of *RhlR* receptor was observed in RBHi when treated with tyrosol. This shows that farnesol and tyrosol actively reduce the expression of the synthase protein (*LasI* and *RhlI*) and prevent production of N-3-oxo-dodecanoyl homoserine lactone (3OC12-HSL) and C4-HSL respectively. *lasR* gene is linked to *lasI* and is considered to be the cognate receptor for 3OC12-HS. Signal synthase, *RhlI*, generates C4-HSL, and the C4-HSL receptor is called *RhlR* (Muh et al. 2006). Significant down-regulation in gene expression was observed with the use of farnesol and tyrosol for exo-proteins *toxA, aprA* and *LasB* as well as *rhlAB* which are responsible for the production of rhamnolipid.

Since the introduction of antibiotics as novel treatment against infections in the 1940s, multi drug resistance traits have become synonymous with biofilm forming opportunistic pathogens that dominate the nosocomial setting (Bryers 2008). This necessitates the identification and investigation of novel antimicrobial therapies and their mode of action. Exploiting interspecies and interkingdom interactions/ competition as well as naturally occurring products and their use in synergy could provide a potential solution to combat/ eradicate biofilm related chronic infections and drug resistance.

### Conclusions

This study demonstrated the effect of quorum quenchers derived from *C. albicans* and the subsequent changes in gene expression of biofilm formation and virulence factors in *P. aeruginosa*.

Our findings have shown promise in identifying several suppressive regulatory effects of *C. albicans* QS signal molecules on AHL mediated *P. aeruginosa* QS network and related virulence factors. It is well known that conventional antibiotics cause resistance in bacteria. This study provides a novel option for the control of *P. aeruginosa* biofilm related infections and diseases, as well as a foundation for future research on the mechanisms of biofilm inhibition in different *P. aeruginosa* phenotypes from the perspective of QQ. The QQs used in this study provide the opportunity for application of these molecules as an alternative to traditional antibiotics. Future challenges for the research include studying the effects of the QQs in vivo. This brings the knowledge obtained from this research closer to real-life application.

Further research will extend the understanding of mechanistic action of the QQ.

Quantification of *P. aeruginosa* virulence factors such as rhamnolipid, pyoverdine, pyocyanin, and elastolytic activity, as well as the use of the crystal violet method to measure biofilm formation upon treatment with quorum quenchers, may help to improve the findings.

## Electronic supplementary material

Below is the link to the electronic supplementary material.

Supplementary Material 1
