# Supplementary material for: Effect of quorum quenchers on virulence factors production and quorum sensing signalling pathway of non-mucoid, mucoid, and heavily mucoid Pseudomonas aeruginosa
Source: World J Microbiol Biotechnol. 2022 Jul 15;38(9):163. doi: 10.1007/s11274-022-03339-9 (PMC9283346; doi:10.1007/s11274-022-03339-9)
Supplement: Supplementary file 1 — Supplementary Material 1 [file 11274_2022_3339_MOESM1_ESM.docx]

**Supplementary Information**

**Fig. S1** Log 10 CFU/mL cell counts of *P. aeruginosa* sp with and without treatment using furanone, farnesol and tyrosol.
